# Developing a powerful *In Silico *tool for the discovery of novel caspase-3 substrates: a preliminary screening of the human proteome

**DOI:** 10.1186/1471-2105-13-14

**Published:** 2012-01-23

**Authors:** Muneef Ayyash, Hashem Tamimi, Yaqoub Ashhab

**Affiliations:** 1 Biotechnology Research Centre, Palestine Polytechnic University, PO-Box: 198, Hebron, Palestine

**Keywords:** Apoptosis, Caspase-3, Caspase substrates, Cleavage site prediction, Position-Specific Scoring Matrix (PSSM), Human proteome, Bioinformatic tool, Pattern recognition

## Abstract

**Background:**

Caspases are a family of cysteinyl proteases that regulate apoptosis and other biological processes. Caspase-3 is considered the central executioner member of this family with a wide range of substrates. Identification of caspase-3 cellular targets is crucial to gain further insights into the cellular mechanisms that have been implicated in various diseases including: cancer, neurodegenerative, and immunodeficiency diseases. To date, over 200 caspase-3 substrates have been identified experimentally. However, many are still awaiting discovery.

**Results:**

Here, we describe a powerful bioinformatics tool that can predict the presence of caspase-3 cleavage sites in a given protein sequence using a Position-Specific Scoring Matrix (PSSM) approach. The present tool, which we call CAT3, was built using 227 confirmed caspase-3 substrates that were carefully extracted from the literature. Assessing prediction accuracy using 10 fold cross validation, our method shows AUC (area under the ROC curve) of 0.94, sensitivity of 88.83%, and specificity of 89.50%. The ability of CAT3 in predicting the precise cleavage site was demonstrated in comparison to existing state-of-the-art tools. In contrast to other tools which were trained on cleavage sites of various caspases as well as other similar proteases, CAT3 showed a significant decrease in the false positive rate. This cost effective and powerful feature makes CAT3 an ideal tool for high-throughput screening to identify novel caspase-3 substrates.

The developed tool, CAT3, was used to screen 13,066 human proteins with assigned gene ontology terms. The analyses revealed the presence of many potential caspase-3 substrates that are not yet described. The majority of these proteins are involved in signal transduction, regulation of cell adhesion, cytoskeleton organization, integrity of the nucleus, and development of nerve cells.

**Conclusions:**

CAT3 is a powerful tool that is a clear improvement over existing similar tools, especially in reducing the false positive rate. Human proteome screening, using CAT3, indicate the presence of a large number of possible caspase-3 substrates that exceed the anticipated figure. In addition to their involvement in various expected functions such as cytoskeleton organization, nuclear integrity and adhesion, a large number of the predicted substrates are remarkably associated with the development of nerve tissues.

## Background

Caspases are a family of intracellular cysteinyl aspartate-specific proteases that are highly conserved in multicellular organisms and are key regulators of apoptosis initiation and execution. At least 14 members of the caspase family have been identified in mammals and they are grouped into two major sub-families, namely inflammatory caspases and apoptotic caspases. Apoptosis associated caspases can be further classified into two groups: initiator caspases, including caspase-2, -8, and -9, which are present upstream of apoptosis signalling pathways; and executioner (effectors) caspases -3, -6, and -7 [1-3].

Initiator capsases-8 and -9 are activated through an auto-cleavage process that is mediated by large adaptor-caspase complexes known respectively as the death-inducing signalling complex (DISC) and apoptosome. These complexes are usually formed in response to an intrinsic or extrinsic cell death stimulus [4]. The main targets of the activated initiator caspases are the executioner procaspases. It is interesting to notice that substrates of initiator caspases are limited to their own precursors, executioner procaspases-3, -6, and -7, and few more proteins [5]. On the other hand, executioner caspases target a large number of cellular proteins to control the dismantling process of the cell [6]. In addition to their essential role in apoptosis, recent accumulated evidence demonstrates various non-apoptotic functions of executioner caspases including: regulation of the immune response, cell proliferation, differentiation and motility [7,8].

Caspases are characterized by high substrate selectivity. They recognize a specific sequence signal in their target proteins. Resolving substrate specificity for caspases was initially investigated using a combinatorial approach with positional scanning of synthetic tetrapeptidyl-aminomethyl coumarin derivatives. The results of this approach determined the absolute requirements for aspartic acid at position P1 [9,10]. In addition, P2 to P4 positions demonstrate high preference for certain amino acids. Based on positional scanning of synthetic tetrapeptides, the preferred recognition sequences for caspases -1, -4, and -5 were determined to be (W/L)EHD, whereas caspases -3, and -7 recognize the sequence DEVD, while caspases- 8, -6, -9, and -10 recognize the sequence (D/L)E(H/T)D.

It is important to emphasize that the *in vitro *caspase substrate specificity, determined by the synthetic tetrapeptide method, is not absolutely representative of the cleavage conditions *in vivo*. The cleavage specificities of caspases *in vivo *are influenced by sequence-dependent conformational features, flanking the cleavage tetrapeptide motif, which can control the molecular electrostatic potential and the steric accessibility of the enzyme to its target protein. For example, and in spite of their identical preference for the DEVD tetrapeptide cleavage motif, caspase-3 and 7 show a clear differential preference for various natural substrates [11,12]. Demon *et al *[13] demonstrated that in addition to the tetrapeptide cleavage core, DEVD (P4-P1), several amino acid positions located outside this core such as P6, P5, P2' and P3' are critical in the discrimination of caspase-7 and caspase-3 for their specific substrates.

Shen *et al *[14] reported another interesting example which proves that the relatively similar tetrapeptide cleavage motif of caspase-1 and caspase-9, which are functionally distinct, does not imply a similar recognition preference for their natural substrates. Via a thorough statistical analysis of a window size of P10-P10' for a collection of caspase-1 and caspase-9 natural substrates, Shen *et al *have determined the significance of various amino acids and/or certain physiochemical properties at certain positions outside the canonical tetrapeptide motif [14].

Among executioner caspases, caspase-3 is considered the major enzyme with a wide array of cellular substrates. While immunodepletion of caspase-3 abolishes the majority of proteolytic events observed during apoptosis, immunodepletion of other executioner caspases shows a minimal impact on apoptosis markers and its proteolytic cleavage outcomes [11]. In the last decade, extensive research on caspases led to the identification of more than 200 caspase-3 substrates and the list is still growing. With the increasing number of proteins that have been discovered, thanks to the sequencing of the human genome and the genomes of many other organisms, there is a need for efficient methods that can help in discovering new caspase-3 substrates. The identification of new cellular substrates for caspase-3 would lead to further insights into the cellular mechanisms that regulate apoptosis, proliferation, and other biological processes.

Bioinformatics tools would allow high-throughput analyses of proteomic data in order to screen for putative caspase-3 substrates. In addition, such tools can provide researchers with an accurate map of the potential cleavage site(s) for a given sequence of interest. In the last few years, several computer-based tools were developed with the aim of predicting caspase substrates. Prediction of Endopeptidase Substrates (PEPS) [15] was among the initial tools and it was developed in order to predict putative caspase-3, cathepsin B and cathepsin L cleavage sites using cleavage site scoring matrices (CSSM). PeptideCutter [16] is another tool that was designed with the objective of predicting cleavage sites for a wide range of proteases including various caspases. GraBCas [17] is a tool that uses a position specific scoring matrix for caspases 1-9 and granzyme B, based on substrate specificities that were determined by positional scanning of synthetic peptides. CaSPredictor [18] was developed based on the assumption that sequences rich in the amino acids Ser (S), Thr (T), Pro (P), Glu or Asp (D/E) (collectively called PEST) are favoured caspase cleavage sites. CaSPredictor was built based on 137 experimentally verified natural substrates for caspase-1, -2, -3, -6, -7, -8, -9, and -10.

In addition to the previously aforementioned scoring-matrix based approaches, several groups recently reported the development of tools that were mainly built up using the support vector machine (SVM) technique. Wee *et al. *[19] described an SVM based approach (called CASVM) using 195 substrates for different caspases from various organisms. Cascleave [20] is an interesting tool that was recently developed utilizing primary sequence, as well as secondary structure features, of the cleaved sites based on SVM approach. Cascleave was built using a dataset of 370 substrates of the different caspases. Piippo *et al *[21] described another tool (termed Pripper) using three different pattern recognition classifiers, namely: SVM, a decision tree based method known as J48, and the Random Forest classifier. The three classifiers were trained on 443 different caspase cleavage sites. Li *et al *[22] proposed a hybrid SVM-PSSM method based on an extended dataset. Unfortunately, some of these tools are not available for testing and comparison purposes.

Despite the substantial efforts to develop *in silico *systems to predict sites of caspase cleavage, the accuracy of such tools is still a challenging issue. The major drawback of the early tools is the use of training datasets that represent synthetic peptides or limited natural substrates for various proteases including caspases. On the other hand, the recently developed SVM-based tools were built using a mixture of heterogeneous data that represent cleavage sites of the different caspases including non-canonical sites as well as some unverified caspase substrates. In general the SVM-based tools such as Cascleave achieve good levels of sensitivity, yet they suffer from high rates of false positive results. It is generally expected that training a prediction tool on data representing distinctive patterns can lead to overgeneralization and hence a high rate of false positive results. It is important to recall that although different caspases share the primary sequence-requirement to cleave at the carboxyl terminal of aspartate residues in their protein targets, each one of these proteases recognizes a unique context surrounding the cleavage position. Even the caspases that appear to have identical tetrapeptide cleavage specificities such as caspase-3 and -7 are actually distinct in terms of the amino acids preferences outside the tetrapeptide core sequence [13]. Based on this assumption, we decided to develop a prediction tool focusing on data that represent substrates of a single caspase. Caspase-3 was selected for this objective as it represents the major executioner caspase with a considerable number of substrates.

In this work, we present a novel tool designated Caspase Analysis Tool 3 (CAT3), which was developed based on an extensive and highly curated dataset of caspase-3 substrates. CAT3 showed an obvious improvement in the overall prediction accuracy as well as a marked reduction of the false positive rate. Using CAT3, a high-throughput screening was performed on a large set of human proteins with assigned Gene Ontology (GO) annotations. The screening results reveal the existence of a large number of potential caspase-3 substrates.

## Implementation

### Methods

#### Caspase-3 substrates

The PubMed literature database [23] was used to search for papers that describe human, mouse, and rat caspase-3 substrates. Each paper was critically analyzed to determine the experimentally demonstrated cleavage position in the relevant protein. The confirmed caspase-3 substrates were 227 proteins with a total of 267 cleavage sites. The amino acid sequences of the proteins were then obtained from the Universal Protein Resource Knowledgebase (UniProtKB) [24]. Of the 267 cleavage sites, 17 sequences were sorted randomly to be used later to compare the performance of CAT3 versus existing similar tools; the remaining 250 sequences, which we refer to as the positive (+) peptide data, were used for training and validation of the CAT3.

#### Definition of study controls

The following datasets were established as controls in this study:

##### The negative uncleaved peptides

This data set consists of all the peptides that contain aspartic acid residues and are presumed to be uncleaved. This control group was established based on the assumption that any D residue, in a caspase-3 substrate, apart from the mapped cleavage site(s) is most likely uncleaved. After excluding the positive peptides that exist in training data, the remaining 8968 D residues were used to create this negative (-) control.

##### Amino acids natural frequency

This control represents the frequency of each one of the 20 amino acids in a group of 20,224 human proteins that were available as reviewed proteins in the UniProtKB [24] as of March 2011.

##### Amino acid R-groups frequency

This control represents the frequencies of the different R-groups of amino acids; acidic (D and E), basic (H, K and R), polar (N, Q, S, T and Y), and non-polar (A, L, P, M, G, V, I, F, W and C). The frequencies were calculated based on the above mentioned 20,224 reviewed proteins.

#### Physiochemical characteristics flanking the cleavage site

The positive peptide sequences were aligned in reference to the cleaved aspartic acid residues. The resulting multiple sequence alignment was divided into three regions: the central tetrapeptide cleavage motif (P4P3P2P1), the N-terminal region preceding the motif and the C-terminal region following the motif that was designated "before-motif" and "after-motif", respectively.

**Figure 1:**
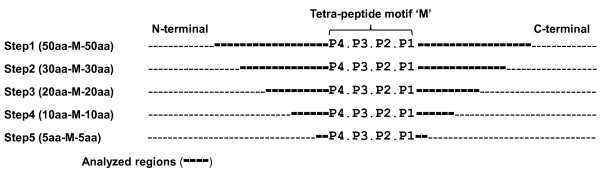
**Sequence analyses**. This drawing shows the regions surrounding the tetra-peptide motif (P4P3P2P1) that were included in the physiochemical analyses. In each step, a given length of amino acids (bold dashed lines) at both N- and C- directions were analyzed.

The analyses for the regions flanking the motif were made serially: 50, 30, 20, 10, and 5 amino acids before and after the motif (Figure 1). The analysis included: the frequencies of amino acids represented by their R-groups (acidic, basic, polar and non-polar), the frequencies of hydrophobic and hydrophilic amino acids and finally the frequencies for each single amino acid. In the case of tetrapeptide motif analysis, the different frequencies were calculated separately for each position: P4, P3, P2 and P1. However, the frequencies within 50, 30, 20, 10, and 5 amino acids, before and after the motif, were calculated collectively for each region.

#### Establishment of scoring matrices

The peptides that fulfil the length criterion, P9-P5', which means having 8 amino acids before and 5 amino acids after the aspartate residue of interest, were used to build the scoring matrices. Both the positive and the negative peptide data sets were used to build the scoring matrices. The first step was to generate position specific frequency matrices from the multiple sequence alignments of the relevant set of peptides. Each matrix consisted of 14 rows, representing positions P9P8P7P6P5 **P4 P3 P2 P1**P1' P2' P3'P4'P5', where a D amino acid is at the position P1. The 20 columns of the matrix represent the frequencies of each amino acid.

From the multiple sequence alignment of the positive peptides, we noticed the presence of two possibly different patterns; the first pattern has a D at P4 (P9...P5-D-X-X-D...P5') and the second has any amino acid except D at P4 (P9...P5-X-{D}-X-X-D...P5'). To represent this subtle difference, we decided to construct amino acid frequency matrices to represent each sub-pattern.

Two weighting systems were used in order to correct the probability of overrepresented and underrepresented amino acids in the frequency matrices so as to establish the scoring matrices:

i) Calculating log odd ratio: This weighting system involves calculating log odd ratio for each element in the frequency matrix by dividing the observed frequency of a given amino acid over its corresponding natural frequency (see the definition of study controls above).

ii) Subtraction of negative control background: Instead of relying only on the common log odd weighting system and in order to minimize scoring bias, we decided to add a second normalization approach. The method relies on comparing the positive peptides with the negative peptides to further remove the noise signals around the cleaved aspartate residues.

Four scoring matrices are involved in the overall calculation of the final score of CAT3 tool. We propose the following notation to define each scoring matrix and the overall score. First, let FM1^+ ^denote the frequency matrix that was constructed from all the positive (+) peptides. The corresponding scoring matrix **'A' **is defined as:

(1) $$A={\text{log}}_{2}\frac{\text{FM}1^{+}}{\Omega }$$

where Ω is the natural frequencies of the amino acids.

In addition to the above scoring matrix we define FM1^- ^as the frequency matrix generated from the negative (-) peptides. A new frequency matrix **'B' **is defined as:

(2) $$B={\text{log}}_{2}\frac{\text{FM}1^{+}-\text{FM}1^{-}}{\Omega }$$

Let ${\text{FM}}_{c}^{\left[\cdot \right]}$ denote a frequency matrix calculated from a subset of peptides that fulfil the constraint **'c'**. Here, [·] is either + or - as explained before.

Therefore, we define the following scoring matrices:

(3) $$C1={\text{log}}_{2}\frac{\left[\text{FM}1_{{}^{\text{P}4=\text{D}}}^{+}\right]-\left[\text{FM}1_{{}^{\text{P}4=\text{D}}}^{-}\right]}{\Omega }$$

and

(4) $$C2={\text{log}}_{2}\frac{\left[\text{FM}1_{{}^{\text{P}4\neq \text{D}}}^{+}\right]-\left[\text{FM}1_{{}^{\text{P}4\neq \text{D}}}^{-}\right]}{\Omega }$$

### CAT3 implementation and scoring

CAT3 tool was built using Perl language. The input protein can be entered either as a FASTA format sequence or as a text file. Once a P14 peptide with a D residue at P1 is identified, it is analyzed to calculate the final score **'S' **as follows:

(5) $$S=\frac{\text{a}+\text{b}+\text{c}}{3}$$

where **'a' **and **'b' **are scores generated from the scoring matrices **'A' **and **'B' **in Equation 1 and Equation 2, respectively. The **'c' **score is generated either from the scoring matrix **'C1'**or **'C2' **as follows:

(6) $$c=\left\{\begin{array}{cc} C1 & \text{if}\;\text{P}4=\text{D}\\ C2 & \text{if}\;\text{P}4\neq \text{D} \end{array}\right)$$

We refer to the scoring matrix **'C1' **if the peptide contains the amino acid D at P4 or the scoring matrix **'C2' **if the amino acid at P4 is not D. The three scores (a, b, and c) are normalized to a 100% score by dividing each score by the maximum score that could be obtained from each formula.

### CAT3 validation

To examine the prediction power of CAT3 a k = 10 fold cross validation was performed. The positive data were the actual cleavage sites, whereas the negative data were obtained from the uncleaved dataset. In each fold four PSSM matrices were created from 9/10th of the positive substrates. Then, the remaining 1/10th positive and negative substrates are used for testing. Since the number of the negative peptides was much larger than the positive peptides, an equal number of the negative peptides were randomly obtained. The whole 10 fold cross validation experiment was repeated 10 times to ensure a good coverage of the negative dataset. The sensitivity (SEN), specificity (SPE), positive predictive value (PPV), negative predictive value (NPV), accuracy (ACC) and the Matthew's correlation coefficient (MCC) were calculated as in [25].

The areas under the receiver operating characteristic (ROC) curves were calculated by plotting the sensitivity against the corresponding 1-specificity. The optimal cut-off point was defined as that measurement that corresponded to the point on the ROC curve closest to the top left corner, i.e., closest to having sensitivity = 1 and specificity = 1.

### Local window size

The most appropriate local window size of amino acid sequence encompassing the cleaved aspartate and other critical exosite residues was determined based on comparing the prediction performance of the following peptides: P3-P1, P4-P1, P5-P1', P6-P2', P7-P3', P8-P4', P9-P5', P11-P7', P14-P10', P17-P13', P20-P16', P23-P19'. The performance of each window size was evaluated using the same cross validation approach as mentioned above. The obtained area under curve (AUC) and Matthew's correlation coefficient (MCC) for the different experiments were plotted for comparison purposes.

### Performance comparison

A performance comparison was carried out for CAT3 versus two recently published prediction tools, namely CASVM and Cascleave [19,20]. The aim of the test was to assess how accurate the three tools were in predicting caspase-3 cleavage sites. The comparison was made on 16 caspase-3 substrates that were randomly excluded from the training dataset.

Since CAT3 is a prediction tool specific for caspase-3 cleavage sites, whereas CASVM and Cascleave were developed to predict cleavage sites of different caspases, there is a possibility to misjudge true positive sites of other caspases by assigning them to the false positive category of CASVM and Cascleave. To avoid such unfair comparison, the 16 substrates were carefully inspected to find all caspase cleavage sites. The search was performed using the PubMed database, Google searching engine, the Caspase Substrate database Homepage (CASBAH) [26], and MERPOS - the Peptidases Database [27].

The protein sequences of the 16 substrates were analyzed individually and the prediction results for each tool were counted according to software default parameters. The true positives are the positively predicted caspase-3 cleavage sites, whereas the false positives are the positively predicted aspartates that are actually not recognized by any caspase.

### High-throughput screening

The UniProtKB [24] was used to retrieve human proteins with known biological processes. Two filters of the advance search option were used: the first was organism: Homo sapiens (Uniprot ID: 9606) and the second was Gene Ontology GO: biological process (GO ID: 0008150). After excluding the experimentally verified 215 human caspase-3 substrates, a total of 13066 reviewed human proteins with defined Gene Ontology (biological process) were obtained. The protein sequences were analyzed by CAT3 to screen for potential novel caspase-3 substrates. Only results of scores ≥ 45 were considered for further analyses. Proteins that were predicted as potential caspase-3 substrates were further analyzed using ToppGene Suite tool [28] to retrieve the most significant Gene Ontology (GO) terms.

## Results

### Caspase 3 substrates

Our search in the PubMed literature database for caspase-3 substrates revealed the presence of 227 proteins: 215 of human origin, 9 of mouse origin, and 3 of rat origin. All the substrates were experimentally verified as natural substrates and their cleavage sites were mapped. Of the 227 substrates, the cleavage sites of 189 proteins were mapped by site-directed mutagenesis technique, while the remaining 38 were mapped by different high-throughput proteomic screening approaches. The full list and description of the obtained substrates are available in the additional materials (Additional file 1). The obtained caspase-3 substrates as well as other caspase substrates will be available in the Caspase Substrates Comprehensive Database (CaspoSome Database) that has been developed at our institute (unpublished results).

### Tetrapeptide cleavage motif analysis

**Figure 2:**
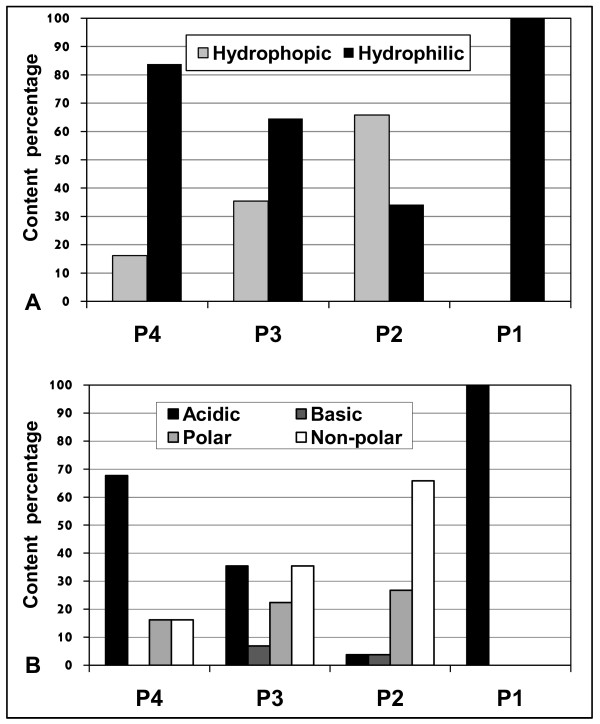
**Physiochemical properties of the tetrapeptide motif**. The content analysis for each position in the tetrapeptide motif (P4P3P2P1) was made for all the cleaved substrates. a) Hydrophobic and hydrophilic amino acid frequencies. b) Acidic, basic, polar and non-polar amino acid frequencies.

The tetrapeptide cleavage motifs (P4P3P2P1) of the training group were analyzed to determine physiochemical properties and frequencies of amino acids at each position. The examination of amino acid frequencies within the tetrapeptide motif revealed a unique distribution pattern of hydrophobic and hydrophilic amino acids (Figure 2.A). Hydrophilic amino acids are 8.6 times more frequent in P4 than hydrophobic amino acids. Interestingly, P3 has an opposite pattern to P2. In P3 hydrophilic amino acids are nearly two times more frequent than hydrophobic amino acids, whereas in P2 the converse is true.

Figure 2.B shows the results of analyzing the frequencies of acidic, basic, polar and non-polar amino acids. In addition to the obvious difference in amino acid group distribution between the four positions and the corresponding control, it is important to notice the lack of basic amino acids in P4 and the high frequency of acidic amino acids in P3 compared to the control.

### Features surrounding the cleavage site

The amino acid sequences surrounding the tetrapeptide cleavage motifs were thoroughly analyzed to identify necessary feature(s) for caspase-3 recognition. The analyses include: secondary structure, amino acids physicochemical properties and amino acid composition.

The secondary structure prediction method GOR4 [29] was used to investigate the cleavage motif and its flanking regions for any common secondary structure(s). The analysis of GOR4 results showed that the majority (80%) of the cleaved sites are located within unstructured context, while 18% are located within alpha helical regions, and only 2% are located in beta sheets.

We then analyzed the biochemical properties of amino acids that flank the tetrapeptide cleavage motif to determine amino acids preferences for caspase-3 substrate recognition. No significant differences in the frequencies of acidic, basic, polar and non-polar amino acids between the tested region and the corresponding control group were found when examining 50, 30, 20, and 10 amino acids before and after the tetrapeptide cleavage motif. When testing the region of 5 amino acids before the cleavage motif a slightly higher percentage of acidic amino acids were noticed, while basic and polar amino acids were strongly unfavoured. In the region of 5 amino acids after the cleavage motif, lower percentages of acidic and basic amino acids were noticed (data not shown).

**Figure 3:**
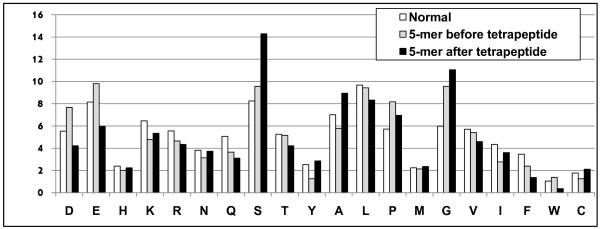
**Amino acid frequencies around the cleavage motif**. The overall frequency of each amino acid was calculated for the two regions: 5-amino acids before (gray bars) and 5-amino acids after (black bars) the tetrapeptide cleavage motif. The observed frequencies were compared to the normal frequency of each amino acid (white bars). Frequencies were obtained as described in the definition of study controls in the Methods' section.

To further explore the characteristic biochemical properties, we examined individual amino acid frequencies for the entire cleavage vicinity: the 5-amino acids before and after the tetrapeptide motif. As shown in Figure 3, the frequencies of glycine, alanine, serine and proline have altered distributions in regions before and after the tetrapeptide motif that may indicate size and charge requirements for caspase-3 recognition and binding to the substrates.

### Position specific scoring matrices

**Figure 4:**
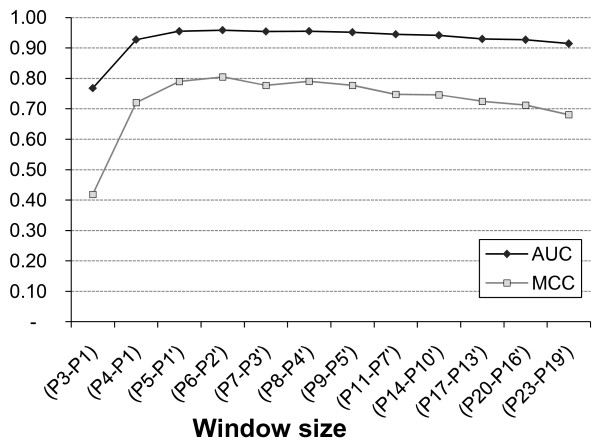
**Window size determination**. Using a 10-fold cross validation, the area under curve (AUC) and Matthew's correlation coefficient (MCC) measures for the indicated window sizes were calculated and plotted to determine the most appropriate local window size.

In order to determine the most appropriate window size to construct efficient scoring matrices for CAT3, a series of gradually increasing window sizes ranging from P3-P1 to P23-P19' were evaluated (Figure 4). As can be seen, it is obvious that window sizes equal to or shorter than the tetrapeptide cleavage motif (P4-P1) are not adequate to develop a reliable prediction tool. Despite of a marginally higher MCC at the window size of P6-P2', the overall prediction efficiency of the window sizes ranging from P5-P1' to P9-P5' are apparently quite similar. However, the efficiency seems to decrease gradually when extending the window size beyond P9-P5'.

We actually preferred the window size P9-P5' over other seemingly comparable shorter alternatives for several reasons. First, the critical analysis of amino acids over-/under-representation scores demonstrated the significance of all the positions in this extended window P9-P5' (Additional file 2). Second, a careful analysis of natural caspase substrates, available in MEROPS database, with cleavage positions near to N- or C- terminals, indicates that minimal adequate N- and C- terminal spacers comparable to the length of P9 and P5', respectively, are required for efficient recognition. Therefore, our scoring matrices were developed by calculating the weight of each amino acid in the 14-mer peptide sequence from P9 to P5'.

To evaluate the contribution of the different amino acids at the positions surrounding the cleaved aspartate, the scoring matrix **'A' **(see Equation 1 in methods section) was drawn as a heat-map (Additional file 2). Analyzing the heat-map shows that apart from the tetrapeptide cleavage motif, all positions have either overrepresented or underrepresented amino acids. However, the positions including P7, P6, P1', P3', P4' and P5' have a remarkable rejection or preference for certain amino acids.

### Prediction power of CAT3

**Figure 5:**
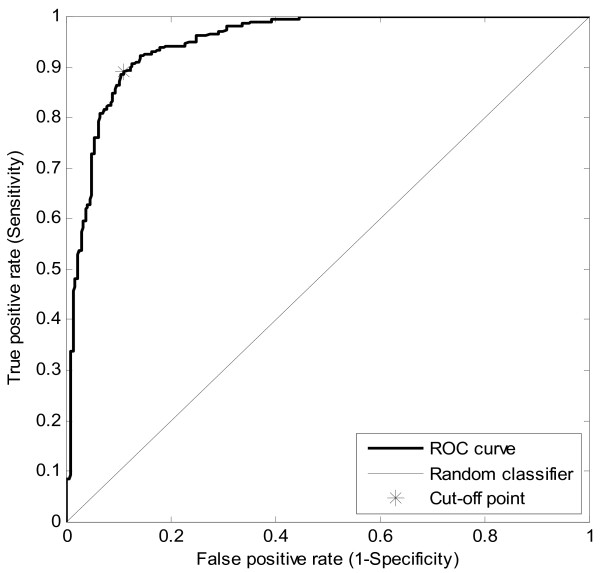
**ROC curve**. Receiver operating characteristic curve (ROC) for CAT3. The curve represents the average of 10 different experiments of the 10-fold cross validation. The X-axis shows the false positive rate, while the Y-axis shows the true positive rate. The asterisk indicates the cut-off point.

A 10 fold cross validation was used to evaluate the predictive power of CAT3. Figure 5 shows the ROC curve that represents the average of 10 different experiments of the 10-fold cross validation. The optimal cut-off score was found to be 30. At this cut-off point the prediction statistical measures are shown in Table 1.

**Table 1:** Cross validation results

Measure	AUC	SPE	SEN	PPV	NPV	ACC	MCC
**Value**	0.9499	0.8850	0.8883	0.8858	0.8886	0.8866	0.7738

The values of the statistical measures represent the average of 10 different experiments of the 10 fold cross validation test. The optimal cut-off score was found to be 30. AUC: Area Under ROC Curve; SPE: Specificity; SEN: Sensitivity; PPV: Positive Predictive Value; NPV: Negative Predictive Value; ACC: Accuracy; MCC: Matthew's correlation coefficient.

To demonstrate specificity of CAT3 for caspase-3 cleavage sites, a group of 25 non-caspase-3 substrates were examined by CAT3. The substrates included 17, 12 and 5 cleavage sites of caspase-1, caspase-8 and caspase-9, respectively. We avoided using any cleavage site that was known to be a shared target with caspase-3. Interestingly, 33 of the 34 cleavage sites (97%) showed CAT3 scores below 30, which is the minimum cut-off for predicting a caspase-3 cleavage site. This result provides a clear evidence to substantiate the very high specificity of CAT3 for predicting caspase-3 substrates. The detailed results of this experiment are available in the additional materials (Additional file 3).

Evaluating the performance of different binary classifiers is frequently made by comparing their reported statistical measures such as specificity, sensitivity etc. which are usually calculated under different conditions. We have avoided the use of such a comparison as it can lead to a biased conclusion. Instead, we compared the performance of CAT3 versus two recently reported tools, namely CASVM and Cascleave, on a group of 16 caspase-3 substrates that were initially excluded from our training data. It is worth mentioning that some of these substrates could have been used in the training of the other tools, which could offer unfair advantage to the other two tools versus CAT3. A thorough examination using different databases revealed that the 16 substrates contain a total of 537 aspartate residues, of which 17 are caspase-3 cleavage sites, 4 are cleavage sites of other caspases and 516 aspartate residues that are evidently not cleaved by any caspase.

**Figure 6:**
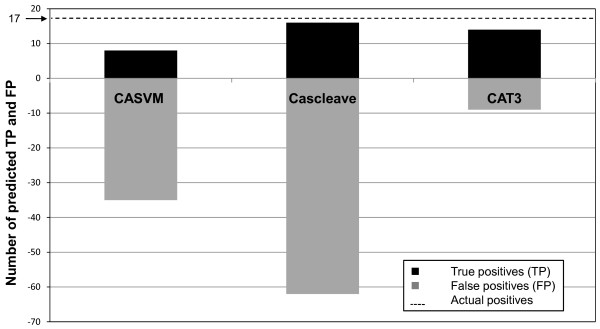
**Performance comparison**. The comparison was made using the tools' default parameters on 16 proteins that have a total of 17 actual caspase-3 cleavage sites. The dashed line shows the actual number of caspase-3 cleavage sites. The black bars show the number of true positives predicted by each tool, while the gray bars (minus scale) show the number of false positives predicted by each tool. The known cleavage sites of the other caspases, which were positively predicted by CASVM or Cascleave, were not counted as false positives.

Out of the 17 actual caspase-3 cleavage sites, the predicted true positive results for the three tools were as follows: CAT3 14/17 (82.3%), CASVM 8/17 (47%), and Cascleave 16/17 (94.1%). However, CAT3 was the best tool in reducing the false positive results (false alarm); out of 516 actual uncleaved aspartic acid containing peptide, CAT3 predicted only 9 false positives (1.7%), while CASVM predicted 35 false positive (6.8%) and Cascleave had 62 (12%) false positives. Figure 6 shows the result of comparing CAT3 versus CASVM and Cascleave. It is noteworthy that both CASVM and Cascleave correctly predicted two of the 4 non-caspase-3 cleavage sites. The detailed results of the comparison are available in the additional materials (Additional file 4).

### High-throughput screening for novel caspase-3 substrates

Screening of 13066 reviewed human proteins with ascribed Gene Ontology (biological process) using our CAT3 tool showed that 3320 proteins are predicted to be caspase-3 substrates with a total of 4903 potential caspase-3 cleavage sites (Additional file 5). To further investigate the function of these potential substrates we used ToppFun: an annotations based gene list functional enrichment analysis tool [28]. Out of the 3320 genes only 3013 had annotations in ToppFun. The analysis revealed a group of 308 biological processes that showed significant enrichment in predicted proteins versus the whole human genome as a control (Additional file 6 and Additional file 7). A careful analysis of these biological processes was performed to shortlist the most significant biological processes by excluding general roots (parents) and detailed leaves (children) of GO terms. The most significant biological processes are shown in Table 2.

**Table 2:** Significantly enriched GO terms

GO ID	GO Term	P-value	Term in predicted proteins	Term in human genome
GO:0007155	cell adhesion	1.02E-39	322	885
GO:0022008	neurogenesis	3.97E-27	324	1016
GO:0007049	cell cycle	2.71E-26	376	1250
GO:0030030	cell projection organization	6.53E-26	263	776
GO:0009966	regulation of signal transduction	6.37E-23	399	1399
GO:0007010	cytoskeleton organization	9.69E-21	217	641
GO:0051276	chromosome organization	8.07E-20	212	630
GO:0000902	cell morphogenesis	1.35E-19	228	698
GO:0040011	locomotion	4.84E-18	310	1071
GO:0045934	negative regulation of nucleobase, nucleoside, nucleotide and nucleic acid metabolic process	6.98E-16	227	737
GO:0009790	embryo development	9.49E-15	245	830

The GO terms of the listed biological processes were manually filtered, to reduce redundancy, by removing general roots (parents) and detailed leaves (children) of the enriched GO terms that were obtained by ToppFun tool. The *P*-value of each GO term in the predicted caspase-3 proteins was derived by random sampling of the whole genome.

ToppFun was also used to examine the enriched cellular component GO terms. Interestingly, the majority of the predicted proteins are located in different nuclear components, cytoskeleton, cell projection, membrane fraction, cell junction, and extracellular matrix, where most typical apoptotic morphological and biochemical changes are observed. The detailed list of the enriched cellular component GO terms is available in the additional materials (Additional file 8).

## Discussion

In addition to its well known key function in apoptosis, caspase-3 has been shown to play a crucial role in the regulation of various biological processes such as cell differentiation, adhesion, neurodevelopment and neuronal signalling [30-32]. Recognition of caspase-3 substrates is becoming a vital need to understand molecular mechanisms behind many disorders including cancer, autoimmune and neurodegenerative diseases. Currently, most of the known caspase-3 substrates have been identified using *in vitro *proteolytic cleavage assays, coupled with site-directed mutagenesis to determine the exact cleavage position. In recognition of the physiological importance of caspase-3, many labs began to perform high-throughput proteomic screening to identify novel substrates of this major caspase [33-36]. Such techniques are relatively expensive and cumbersome. In addition to the high cost, the number of identified substrates is usually limited to the proteins that are relatively abundant in the examined cell type.

Recently, several computer-based prediction tools such as CASVM, Cascleave, and Pripper were developed in order to help discover novel caspase substrates [19-21]. These tools were trained on data that represent substrates of different caspases and in some cases non-caspase endopeptidase. Although different caspases share a primary sequence-requirement, to cleave at the carboxyl terminal of aspartate residues in their protein targets, each one of these proteases needs a special context surrounding the cleavage position. Even the caspases that appear to have identical tetrapeptide cleavage specificities such as caspase-3 and -7 are actually distinct in terms of the amino acids preferences outside the tetrapeptide core sequence [13]. Therefore, we believe that building a single algorithm for predicting the cleavage of multi-caspases would likely have low prediction specificity. Based on this hypothesis, we decided to develop an algorithm focusing only on substrates of caspase-3, which is the major executioner caspase with a considerable number of targets. Our caspase-3-specific approach (CAT3) has indeed outperformed other multi-caspases prediction tools on an independent comparison-dataset (Figure 6).

CAT3 has three distinctive features. Firstly, it was developed using PSSM instead of other relatively complex approaches. PSSM is known to be practical, require low computation power and is able to represent the statistical weights of amino acids at each position. In addition, it can be easily combined with other machine learning tools to generate hybrid approaches that might enhance the prediction performance.

Secondly, instead of using data for different caspases, which are actually a mixture of heterogeneous patterns, we used an extended set of highly-curated caspase-3 natural substrates. We believe that inclusion of data that represents other proteases or cleavage sites of caspases with very few substrates and/or cleavage positions representing non-canonical patterns can lead to overgeneralization. In this situation, the classification model is required to loosen its decision boundary to increase sensitivity, but at the cost of having more false positive results. It is therefore generally accepted that improvement in prediction accuracy is more likely to be associated with the good quality of the used data rather than the complexity of the classification method. CAT3, indeed, showed a very low rate of false positive results in comparison to existing state-of-the-art tools, namely, CASVM and Cascleave.

Thirdly, CAT3 is a straightforward sequence-based scoring system that offers an easy to use reference scale to determine the potential cleavage site(s) instead of offering a yes-no answer or providing many suggested cleavage sites without any score to rank them. In contrast to other tools that can execute a single sequence per query, CAT3 is a fast system that can process both single and multiple sequence inputs: a feature that would assist biologists to perform large scale *in silico *screening to identify novel caspase-3 substrates.

Our secondary structure analyses of caspase-3 substrates showed that regardless of cleavage patterns, aspartic acid residues are predominantly located in unstructured regions and to a lesser extent within alpha-helices. In addition, we found the amino acids D, E, A, G and S appear more frequently in natively unstructured regions no matter whether they lie within or outside cleavage motifs. These findings are in agreement with various reports that used statistical analysis to determine the natural distribution of these amino acids and their influence on secondary structure [37-39]. This interesting result raises a question about the benefit of using local secondary structure properties of the cleavage sites as additional features to enhance the discrimination between cleaved and noncleaved patterns [20].

Careful evaluation of amino acid preference, at the positions surrounding the tetrapeptide cleavage motif, points to a general trend where the unfavourable amino acids have greater weight than the favoured ones, especially at P7, P6, P1', P3', P4', and P5' (Additional file 2). Nevertheless, P1' has a remarkable preference for specific amino acids, namely glycine and serine. In addition to their role in determining the molecular electrostatic potential and the steric accessibility of the enzyme, the post-translational modification potential of these two amino acids is vital for determining the timing and functional consequences of cleavage. Tözsér *et al. *[40] demonstrated that phosphorylation of serine residues in close proximity to the tetrapeptide cleavage core can determine caspase-3 cleavability. On the other hand, the high preference for glycine at P1' can be crucial to the acquisition of a myristic acid at this residue. Myristoylation is a co-translational reaction that occurs after the removal of the initiator methionine residue. It can also occur as a post-translational modification when internal glycine residues become exposed after caspase cleavage. The addition of a myristate moiety can alter subcellular localization of the cleaved proteins by facilitating their attachment to membranes and other proteins [41].

By using CAT3, we carried out a large scale proteomic screen to identify novel potential caspase-3 substrates. The initial screening showed that 3320 human proteins can be potential caspase-3 substrates. Even after normalizing this result by excluding the noise coming from the presumed false positive rate, the percentage of potential caspase-3 substrates in the human proteome would be roughly ~14%. This means that only a small fraction (less than ~10%) of caspase-3 substrates has so far been discovered.

The results of GO term enrichment analysis using ToppFun showed that the majority of the predicted caspase-3 substrates are involved in cell adhesion, signal transduction, cell cycle, cytoskeleton organization, chromosome organization, neurogenesis, embryo development, cell morphogenesis, DNA metabolism (Table 2). It is interesting to note the direct association of some of these processes to the biochemical events that lead to characteristic morphological changes in an apoptotic cell. These changes include the breakup of the nuclear envelope and actin filaments in the cytoskeleton, blebbing of the plasma membrane, cell shrinkage, nuclear fragmentation, chromatin condensation, and chromosomal DNA fragmentation [42,43].

It is interesting to notice the remarkable presentation of some biological processes that are not related to apoptosis such as cell development and neurogenesis. The careful analysis of the enriched biological processes GO terms demonstrates a possible significant role of caspases-3 in the development and differentiation of nerve cells. In fact, several reports have shown a strong expression of non-apoptotic active caspase-3 in various proliferating and differentiating neuronal cells [44,45]. Further investigation focusing on the role of caspases in nerve tissue may reveal new pathways that are necessary for the development and differentiation of nerve cells.

The results of enriched cellular component GO terms showed that most of the predicted substrates are distributed to nuclear components (nucleoplasm, nucleolus, chromosome, and nuclear envelope), cytoplasmic components (cytoskeleton and cell projection), and plasma membrane part (cell projection and membrane fraction). This distribution is correlated with the normal subcellular localization of caspase-3. Although the procaspase-3 is localized in the cytoplasm, active caspase-3 plays essential roles both in the cytoplasm and nucleus [46]. Feng *et al. *[47] have shown that the activated caspase-3 is first observed close to the inside surface of the cellular membrane, then transferred to the cytoplasm, and finally translocated to the nucleus.

An interesting fraction of the predicted caspase-3 substrates are proteins of the extracellular matrix. The cleavage of such proteins can be achieved through their cytoplasmic embedded domains. Further investigations are needed to shed light on the biological importance of extracellular matrix proteins and their association with apoptotic and non-apoptotic roles of caspases.

## Conclusions

In this work, we introduce a significant improvement to the *in silico *prediction approach of caspase substrates. Based on our results and in order to increase prediction specificity, we suggest the caspase-specific approach instead of that based upon considering the different caspases' substrates as having one common pattern. CAT3 can be considered a prototype system that would be easily utilized in developing prediction tools for other caspases and endopeptidases. The predicted cellular targets of CAT3 might be used to explore new pathways to gain further insight into the cellular mechanisms that regulate apoptosis, proliferation, and other biological processes. In addition, the discovery of such targets might have significant implications for the development of drugs for various diseases including cancer, autoimmune disorders and neurodegenerative pathologies.

## Availability and requirements

**Project name**: CAT3

**Operating system(s)**: Windows

**Programming language**: Perl

**Other requirements**: none

**Any restrictions to use by non-academics**: none

Note: CAT-3 v 1.0 software that can process both single and multiple sequences is provided in the additional materials (Additional file 9).

## List of abbreviations

PSSM: Position Specific Scoring Matrix; AUC: Area Under Curve; ROC: Receiver Operating Characteristic; SVM: Support Vector Machine; UniProtKB: Universal Protein Resource Knowledgebase; GO: Gene Ontology; SEN: Sensitivity; SPE: Specificity; PPV: Positive Predictive Value; NPV: Negative Predictive Value; ACC: Accuracy; MCC: Matthew's Correlation Coefficient

## Authors' contributions

MA and YA jointly performed data collection and verification, MA performed the sequence analysis, wrote the CAT3 Perl code, and helped to draft the methodology section of the manuscript. HT carried out the cross validation experiments, wrote the code to run the high-throughput screening, and helped to draft the validation and implantation sections of the manuscript. YA designed and supervised the study, carried out the cleavage pattern analysis, performed the analysis of the high-throughput screening, and wrote the manuscript. All authors read and approved the final manuscript.

## Supplementary Material

Additional file 1 **Full list of caspase-3 substrates**. This table shows the description of the obtained 227 caspase-3 substrates. The cleavage evidence refers to the experimental method through which cleavage was identified. SDM stands for site directed mutagenesis, while proteomics refers to experiments of high-throughput proteomic screening. Click here for file

Additional file 2 **Heat-map representing caspase-3 cleavage pattern**. This heat-map represents the scores of the 20 amino acids in the scoring matrix 'A' (see establishment of scoring matrices in the Methods' section). The colour intensities reflect the magnitude of amino acid scores. The blue scale denotes the positive scores, while the yellow to red scale denotes the negative values. Click here for file

Additional file 3 **Prediction results of non-caspase-3 substrates**. The three Excel sheets show the prediction results for 25 non-caspase-3 substrates (17 cleavage sites for caspase-1, 12 cleavage sites for caspase-8 and 5 cleavage sites for caspase-9). The Pub Med ID reference and the CAT3 score for each cleavage site are shown. Click here for file

Additional file 4 **Detailed results of the performance comparison**. This table shows the prediction results of CAT3 versus CASVM and Cascleave for 16 caspase substrates (17 cleavage sites) that were not originally included in the training of CAT3. The cleavage position(s) in all these substrates were confirmed by site directed mutagenesis. Click here for file

Additional file 5 **List of predicted potential human caspase-3 substrates**. This table shows the list of 3320 human proteins that were predicted to be caspase-3 substrates. Several proteins have more than one predicted site leading to a total of 4903 potential cleavage sites. Only cleavage sites of scores ≥ 45 are shown. Click here for file

Additional file 6 **List of the significantly enriched biological process GO terms**. This table shows the full list of the significantly enriched terms of GO: Biological Process among the 3013 predicted caspase-3 substrates. Click here for file

Additional file 7 **Chart representing biological processes among predicted substrates**. This figure shows the graphical representation of the enriched GO terms: Biological Process among the 3013 predicted caspase-3 substrates. The red bars indicate the gene count (Y-axis) per each GO term: Biological Process (X-axis). The blue dotted line shows the *p*-value for each GO term that was derived by random sampling from the whole genome analysis. Click here for file

Additional file 8 **List of the significantly enriched cellular component GO terms**. This table shows the full list of the significantly enriched terms of GO: Cellular Component among the 3013 predicted caspase-3 substrates. Click here for file

Additional file 9 **Installation files for CAT-3 tool**. This compressed folder contains a total of 6 files: final_CAT3.exe, process.exe, algmodule.pm, and submodules.pm that are needed for CAT3 software to work on Windows operating system. The two files: Single_sequence-test.txt and Multiple_sequences-test.txt are provided for testing purposes. To install CAT-3 v1.0 on your PC, copy the 6 files to one folder and thereafter you can simply start working by clicking on the CAT3.exe file. Click here for file
