# Supplementary material for: Developing a powerful In Silico tool for the discovery of novel caspase-3 substrates: a preliminary screening of the human proteome
Source: BMC Bioinformatics. 2012 Jan 23;13:14. doi: 10.1186/1471-2105-13-14 (PMC3324375; doi:10.1186/1471-2105-13-14)
Supplement: Additional file 1 — Full list of caspase-3 substrates. This table shows the description of the obtained 227 caspase-3 substrates. The cleavage evidence refers to the experimental method through which cleavage was identified. SDM stands for site directed mutagenesis, while proteomics refers to experiments of high-throughput proteomic screening. [file 1471-2105-13-14-S1.PDF]

Protein Entry name	Tetra-peptide motif	Cleavage position	Cleavage evidence	PubMed-ID (refrence)
DFFA_HUMAN	DETD	117	SDM	9564035
DFFA_HUMAN	DAVD	224	SDM	9564035
DC1I1_HUMAN	DSGD	116	SDM	11425872
APAF_HUMAN	SVTD	271	SDM	11550094
DCLK1_HUMAN	DEND	369	SDM	11479289
B2L11_HUMAN	SECD	13	SDM	14732682
PRKN2_HUMAN	LHTD	126	SDM	11839750
ERBB2_HUMAN	SETD	1125	SDM	11402024
A4_HUMAN	VEVD	739	SDM	10319819
GELS_HUMAN	DQTD	403	SDM	9323209
RB_HUMAN	DSID	349	SDM	11960384
RU17_HUMAN	DGPD	341	SDM	8642305
PARP1_HUMAN	DEVD	214	SDM	8090205
ANDR_HUMAN	DEDD	155	SDM	9886069
BCL2_HUMAN	DAGD	34	SDM	10799576
TOP1_HUMAN	DDAD	146	SDM	9933635
TOP1_HUMAN	EEED	170	SDM	9933635
VDR_HUMAN	DMMD	198	SDM	18832097
CADH1_HUMAN	DTRD	750	SDM	12859942
CEAM1_HUMAN	DQRD	465	SDM	12637508
VAV_HUMAN	DLYD	161	SDM	10713703
ITB4_HUMAN	DELD	1109	SDM	17178732
SPI1_HUMAN	HVLD	97	SDM	19281794
SPI1_HUMAN	GEAD	151	SDM	19281794
ICAL_HUMAN	DAID	233	SDM	10731697
RASA1_HUMAN	DTVD	455	SDM	11463818
RASA1_HUMAN	DEGD	157	SDM	11463818
CD3Z_HUMAN	DTYD	154	SDM	10197606
KS6B1_HUMAN	TPVD	393	SDM	19191576
IF4B_HUMAN	DETD	45	SDM	10672017
AT2B4_HUMAN	DEID	1080	SDM	11751908
APC_HUMAN	DNID	777	SDM	9973322
IKBA_HUMAN	DRHD	31	SDM	15202778
PDE4A_HUMAN	DAMD	72	SDM	10829034
2AAA_HUMAN	DEQD	218	SDM	9582351
DSG3_HUMAN	DYAD	781	SDM	11500511
CTNB1_HUMAN	DLMD	764	SDM	10748026
CTNB1_HUMAN	YPVD	751	SDM	10748026
RFC1_HUMAN	DEVD	723	SDM	9351817
ADDA_HUMAN	DDSD	633	SDM	10823823
SRBP1_HUMAN	SEPD	460	SDM	8605870
BRCA1_HUMAN	DLLD	1155	SDM	12149654
CDN1A_HUMAN	DHVD	112	SDM	9668108
MLH1_HUMAN	DKTD	418	SDM	15087450
LAP2A_HUMAN	DKDD	109	SDM	10318766
HD_HUMAN	DSVD	511	SDM	9535906
DCC_HUMAN	LSVD	1290	SDM	9796814
TNNT2_HUMAN	DFDD	99	SDM	11972044

BAT3_HUMAN	DEQD	1001	SDM	14960581
CDN1B_HUMAN	DPSD	139	SDM	10490817
NEDD4_HUMAN	DQPD	598	SDM	9593687
PA24A_HUMAN	DELD	522	SDM	9875225
GSH1_HUMAN	AVVD	499	SDM	12000740
FNTA_HUMAN	VSLD	59	SDM	11313965
NU153_HUMAN	DITD	349	SDM	10318766
PSN2_HUMAN	DSYD	329	SDM	9353287
APLP1_HUMAN	VEVD	620	SDM	12124429
BMX_HUMAN	DFPD	242	SDM	11278797
ATN1_HUMAN	DSLD	109	SDM	10085113
CASP9_HUMAN	DQLD	330	SDM	12506111
HDAC4_HUMAN	DVTD	289	SDM	15205465
BCAR1_HUMAN	DVPD	318	SDM	10712510
BCAR1_HUMAN	DSPD	650	SDM	10712510
ACTB_HUMAN	ELPD	244	SDM	8990178
PTEN_HUMAN	QEID	301	SDM	12788938
PTEN_HUMAN	DVSD	371	SDM	12788938
PTEN_HUMAN	DTTD	384	SDM	12788938
CDC42_HUMAN	DLRD	121	SDM	11278572
RAC1_HUMAN	VVGD	11	SDM	12897143
RAC1_HUMAN	VMVD	47	SDM	12897143
IF4G2_HUMAN	DETD	792	SDM	10611228
PRKDC_HUMAN	DEVD	2713	SDM	8670824
HNRPU_HUMAN	SALD	100	SDM	10671544
MDM2_HUMAN	DVPD	361	SDM	9840926
SPTB2_HUMAN	DEVD	1457	SDM	9712874
KPCE_HUMAN	SSPD	383	SDM	12198125
KPCE_HUMAN	DDVD	451	SDM	12198125
KPCT_HUMAN	DEVD	354	SDM	9252332
FAK1_HUMAN	DQTD	772	SDM	9642276
KPCZ_HUMAN	EETD	210	SDM	11016947
KPCZ_HUMAN	DGMD	239	SDM	11016947
KPCD_HUMAN	DMQD	329	SDM	15942654
RAD51_HUMAN	DVLD	187	SDM	10998058
MCL1_HUMAN	EELD	127	SDM	15637055
MCL1_HUMAN	TSTD	157	SDM	15637055
DPOE1_HUMAN	DMED	1214	SDM	11058115
PP2BA_HUMAN	DGFD	385	SDM	10898953
DHX9_HUMAN	DTPD	96	SDM	10570320
SSRP1_HUMAN	DQHD	450	SDM	16498457
SRBP2_HUMAN	DEPD	468	SDM	8605870
DLG1_HUMAN	YEVD	756	SDM	19054065
TIAM1_HUMAN	DETD	993	SDM	11751455
STK4_HUMAN	DEMD	326	SDM	9545236
TRAF1_HUMAN	LEVD	163	SDM	11098060
PAK2_HUMAN	SHVD	212	SDM	9786869
STK3_HUMAN	DELD	322	SDM	9545236
ATM_HUMAN	DYPD	863	SDM	10454555

ROCK1_HUMAN	DETD	1113	SDM	11283607
4EBP1_HUMAN	VLGD	25	SDM	11865047
SPTA2_HUMAN	DETD	1185	SDM	9712874
SPTA2_HUMAN	DSLD	1478	SDM	9712874
IL16_HUMAN	SSTD	1211	SDM	9422780
IL18_HUMAN	DMTD	71	SDM	9334240
CASL_HUMAN	DLVD	363	SDM	10866674
CASL_HUMAN	DDYD	630	SDM	10866674
ITPR1_HUMAN	DEVD	1900	SDM	16316991
GOGB1_HUMAN	DVTD	1946	SDM	14970262
GOGB1_HUMAN	DASD	1134	SDM	14970262
KPCD1_HUMAN	CQND	378	SDM	10622742
RABE1_HUMAN	DESD	438	SDM	9321397
PKN1_HUMAN	DFLD	454	SDM	9751706
PKN2_HUMAN	DITD	117	SDM	9368003
PKN2_HUMAN	DEVD	700	SDM	9368003
2A5E_HUMAN	DKVD	15	SDM	20807766
TAOK1_HUMAN	DVSD	376	SDM	16407310
PARG_HUMAN	DEID	256	SDM	11053413
Q86X94_HUMAN	DQPD	109	SDM	19426707
TENS4_HUMAN	DSTD	570	SDM	15806167
AKAP1_HUMAN	DSVD	582	SDM	18497968
AN32B_HUMAN	AEVD	163	SDM	20015864
M4K1_HUMAN	DDVD	385	SDM	10602493
BAD_HUMAN	PAGD	29	SDM	11287608
BIRC7_HUMAN	DHVD	52	SDM	14559822
CDC6_HUMAN	SEVD	442	SDM	14517333
PI51A_HUMAN	DIPD	292	SDM	11042212
GORS1_HUMAN	SLLD	317	SDM	11815631
GORS1_HUMAN	SFLD	372	SDM	11815631
GORS1_HUMAN	TLPD	390	SDM	11815631
MST4_HUMAN	DESD	305	SDM	11741893
HDAC6_HUMAN	DMAD	1088	SDM	19596000
DNJB4_HUMAN	MEID	128	SDM	20494979
STK39_HUMAN	DEMD	392	SDM	10980603
DBNL_HUMAN	EHID	361	SDM	11689006
TAOK2_HUMAN	DPGD	919	SDM	17158878
Q9ULW9_HUMAN	DLFD	315	SDM	11602184
CD11A_HUMAN	YVPD	394	SDM	9115219
CSEN_HUMAN	DSSD	64	SDM	11278424
STK24_HUMAN	AETD	325	SDM	12107159
SNCAP_HUMAN	DEVD	454	SDM	16495229
BECN1_HUMAN	DLFD	124	SDM	21203962
PTMA_HUMAN	DDVD	101	SDM	10623890
PTMA_HUMAN	DEDD	98	SDM	10623890
BECN1_HUMAN	DQLD	149	SDM	21203962
MDC1_HUMAN	EEVD	173	SDM	21148072
MDC1_HUMAN	DSDD	497	SDM	21148072
CND2_HUMAN	DFPD	366	SDM	21151026

TERA_HUMAN	DELD	307	SDM	18596415
TERA_HUMAN	DELD	580	SDM	18596415
ARPC5_HUMAN	DEED	29	Proteomics	20627866
ZC3H1_HUMAN	DTTD	335	Proteomics	20627866
UBP1_HUMAN	DFTD	41	Proteomics	20627866
PCF11_HUMAN	DKTD	413	Proteomics	20627866
CLCA_HUMAN	DAVD	76	Proteomics	20627866
MYH9_HUMAN	DTLD	1153	Proteomics	20627866
MYH10_HUMAN	DTLD	1160	Proteomics	20627866
NASP_HUMAN	DMLD	523	Proteomics	20627866
LRBA_HUMAN	SSVD	1756	Proteomics	20627866
THOP1_HUMAN	DMAD	13	Proteomics	20627866
NFAC3_HUMAN	DDVD	193	Proteomics	20627866
LIPA1_HUMAN	SETD	836	Proteomics	20627866
TADBP_HUMAN	DETD	89	Proteomics	20627866
TIF1B_HUMAN	DGAD	688	Proteomics	20627866
TCOF_HUMAN	DDPD	1242	Proteomics	20627866
IF4H_HUMAN	DEVD	93	Proteomics	20627866
RN219_HUMAN	DFCD	433	Proteomics	20627866
BN3D2_HUMAN	TELD	8	Proteomics	20627866
UBR7_HUMAN	DEYD	336	Proteomics	20627866
DDX1_HUMAN	DEFD	685	Proteomics	20627866
PAWR_HUMAN	EEPD	131	Proteomics	20627866
CQ056_HUMAN	DELD	192	Proteomics	20627866
WIBG_HUMAN	DQPD	143	Proteomics	20627866
NADAP_HUMAN	DSLD	537	Proteomics	20627866
PKRI1_HUMAN	DYMD	97	Proteomics	20627866
GMIP_HUMAN	DTKD	842	Proteomics	20627866
DIP2B_HUMAN	QETD	60	Proteomics	20627866
CING_HUMAN	SSVD	173	Proteomics	20627866
CFDP1_HUMAN	DEVD	40	Proteomics	20627866
DLGP4_HUMAN	DTQD	720	Proteomics	20627866
MOBL3_HUMAN	DEMD	34	Proteomics	20627866
HYOU1_HUMAN	DDVD	338	Proteomics	20627866
ACTB_HUMAN	DSGD	157	Proteomics	19892738
PPM1G_HUMAN	DDVD	138	Proteomics	19892738
PDXD1_HUMAN	DNVD	584	Proteomics	19892738
STX12_HUMAN	DLID	217	Proteomics	19892738
STX7_HUMAN	DVID	204	Proteomics	19892738
ROA1_HUMAN	EEVD	69	SDM	9774422
ROA1_HUMAN	DDHD	157	SDM	9774422
DESP_HUMAN	DVLD	1641	Proteomics	17728405
FUBP1_HUMAN	DQPD	74	Proteomics	17728405
ROA2_HUMAN	AEVD	76	Proteomics	17728405
NUCL_HUMAN	TEID	455	Proteomics	17728405
KTN1_HUMAN	DNAD	241	Proteomics	17728405
LMNB1_HUMAN	VEVD	231	Proteomics	17728405
HS90B_HUMAN	DEED	259	Proteomics	17728405
RBP2_HUMAN	DVTD	111	Proteomics	17728405

LA_HUMAN	DEHD	371	Proteomics	17728405
HNRPQ_HUMAN	DERD	382	Proteomics	17728405
TAXB1_HUMAN	DGAD	607	Proteomics	17728405
PRS7_MOUSE	DEID	278	SDM	20424172
PO121_RAT	DKTD	531	SDM	14729472
ACINU_HUMAN	DELD	1093	SDM	10490026
AKT1_HUMAN	ECVD	462	SDM	12124386
AKT1_HUMAN	TVAD	108	SDM	11463786
AKT1_HUMAN	EEMD	119	SDM	11463786
B3AT_HUMAN	TATD	45	SDM	14570914
B3AT_HUMAN	EQGD	205	SDM	14570914
CCNE1_HUMAN	LDVD	275	SDM	11884622
DSG1_HUMAN	DLRD	888	SDM	16286477
FHOD1_HUMAN	SVPD	616	SDM	17013756
GRAP1_HUMAN	DTVD	598	SDM	12207967
DFFA_MOUSE	DEPD	117	SDM	9108473
DFFA_MOUSE	DAVD	224	SDM	9108473
KU86_HUMAN	DLLD	730	SDM	16002990
PSIP1_HUMAN	EVPD	30	SDM	12181742
PSIP1_HUMAN	WEID	85	SDM	12181742
PSIP1_HUMAN	DAQD	486	SDM	12181742
MITF_HUMAN	DLTD	345	SDM	16140982
PDE4A_MOUSE	DAVD	72	SDM	10829034
PTN12_MOUSE	DSPD	552	SDM	17130234
PAXI_HUMAN	NPQD	102	SDM	11825902
PAXI_HUMAN	SQLD	301	SDM	11825902
PAXI_HUMAN	DDLD	5	SDM	11825902
PAXI_HUMAN	SLLD	222	SDM	11825902
KPCE_MOUSE	SATD	383	SDM	9431985
AT2B2_HUMAN	EEID	1117	SDM	12107825
TEBP_HUMAN	PEVD	142	SDM	16038904
RET_HUMAN	VSVD	707	SDM	10921886
RET_HUMAN	DYLD	1017	SDM	10921886
SRC_HUMAN	SLHD	502	SDM	10775506
TPR_HUMAN	DSQD	1892	SDM	11423910
TPR_HUMAN	DGTD	2013	SDM	11423910
TPR_HUMAN	DGND	1999	SDM	11423910
STAT1_HUMAN	MELD	694	SDM	9535846
TRAF3_HUMAN	EEAD	348	SDM	11261798
TRAF3_HUMAN	ESVD	368	SDM	11261798
TWST1_MOUSE	DELD	173	SDM	16096654
BIRC4_HUMAN	SESD	242	SDM	10508158
STX5_RAT	DMMD	249	SDM	14970262
STX5_RAT	DEQD	263	SDM	14970262
BLM_HUMAN	TEVD	415	SDM	11154689
PDE6A_HUMAN	DFVD	167	SDM	11602184
MDM4_MOUSE	DVPD	361	SDM	11840332
MAGD1_HUMAN	SEPD	222	Proteomics	20627866
MAGD1_HUMAN	DALD	678	Proteomics	20627866

VIME_HUMAN	DSVD	85	SDM	11423904
VIME_HUMAN	CEVD	331	SDM	11423904
SRP54_HUMAN	DSTD	391	Proteomics	20627866
NUMA1_HUMAN	DSLD	1726	SDM	12508117
IF4G1_HUMAN	DRLD	1175	SDM	10889507
PLCG1_HUMAN	AEPD	770	SDM	10834929
BAG3_HUMAN	KEVD	347	SDM	20232307
BUB1B_HUMAN	DTCD	610	SDM	16227576
EIF3J_HUMAN	DLAD	242	SDM	15900314
FYN_HUMAN	EERD	19	SDM	10435619
GAS2_HUMAN	SRVD	278	SDM	10564664
GRAP2_HUMAN	DIND	241	SDM	11391000
IF2A_HUMAN	AEVD	301	SDM	10432301
IL33_MOUSE	DGVD	175	SDM	20035719
K1C18_HUMAN	VEVD	238	SDM	9298992
K1C18_HUMAN	DALD	397	SDM	9298992
LMNA_HUMAN	VEID	230	SDM	8978814
LYN_HUMAN	DGVD	18	SDM	11526478
MDM4_HUMAN	DVPD	361	SDM	11840332
MET_HUMAN	ESVD	1002	SDM	15542841
PA2G6_HUMAN	DVTD	183	SDM	10747887
RAD21_HUMAN	DSPD	279	SDM	11875078
SRP72_HUMAN	SELD	614	SDM	9857079
