# Supplementary material for: Developing a powerful In Silico tool for the discovery of novel caspase-3 substrates: a preliminary screening of the human proteome
Source: BMC Bioinformatics. 2012 Jan 23;13:14. doi: 10.1186/1471-2105-13-14 (PMC3324375; doi:10.1186/1471-2105-13-14)
Supplement: Additional file 2 — Heat-map representing caspase-3 cleavage pattern. This heat-map represents the scores of the 20 amino acids in the scoring matrix 'A' (see establishment of scoring matrices in the Methods' section). The colour intensities reflect the magnitude of amino acid scores. The blue scale denotes the positive scores, while the yellow to red scale denotes the negative values. [file 1471-2105-13-14-S2.PDF]

	P9	P8	P7	P6	P5	P4	P3	P2	P1	P1'	P2'	P3'	P4'	P5'
A	0.539	-0.3365	0.4055	0.8938	-0.6419	-1.1527	0.069	-0.2412		0.3285	0.5306	1.2879	0.5341	-0.0953
C	0.3365	-0.5108	0.5108	1.0986	0.4055	-0.5108	-0.6931	-0.6931		0.47	-0.5596	1.5041	-0.5108	0.2231
D	0.3102	0.3448	-0.1252	0.2513	0.5798	1.9752	-0.6131	-1.2993	D	-0.5596	-0.5108	-0.1823	-1.0116	0.1001
E	0.2877	-0.2412	0.077	-0.3567	0.5754	0	1.3863	-1.5261		-1.7047	-1.0647	0.3285	0.2136	0.2578
F	-0.5878	0.6931	-0.3185	0	1.0116	-2.5649	-0.3677	-0.1823		0.1178	-0.6931	-0.6931	-0.6931	-1.2993
G	0.4308	0.7985	1.1527	0.3228	0.1335	-1.8718	-0.4055	-0.3365		1.3322	0.5705	0.6931	0.6131	-0.2624
H	-0.1178	0.4055	0.2877	-1.7918	-0.6931	-1.5041	-0.5108	-0.1542		-0.3365	0.6931	-0.5108	0.3365	0
I	0	0.08	-0.452	-1.0986	-0.1942	-2.3979	-1.6094	0.5108		-0.8873	-0.2877	-0.3677	-0.3677	0.2231
K	-0.1178	-0.1823	0.069	-0.7985	-0.4855	-3.0445	-0.7732	-2.2513		-1.0986	-0.1466	-0.383	0.3054	0
L	0.2451	-0.0953	-0.1178	0	-0.1603	-1.335	-0.5341	0.1942		-0.8755	-0.1335	-0.2048	-0.4055	-0.2336
M	0.3365	-0.5596	0.2231	-0.5108	0.2877	-0.8473	0.3567	0.6061		-1.9459	0.6931	0.9163	1.204	-1.8718
N	-0.7538	0.4855	-1.7346	-0.4855	-0.3185	-1.8718	-0.8109	-0.5596		0.087	-0.619	-0.6061	-0.3365	-0.1823
P	-0.2113	0.1911	0.6419	1.0498	0.1671	-1.5404	-0.6061	0.9694		-0.9163	0.1942	0.1942	0.4418	0.4274
Q	0	-0.2513	-0.636	0.2231	0.1942	-0.9808	0	-0.4418		-1.8718	0.3677	-0.6931	-0.6931	0.4353
R	-0.2513	-0.6931	0.3795	-0.2231	-0.3677	-2.3979	-0.9808	-0.8473		-1.204	0.3567	-0.1942	-0.3365	-0.1671
S	-0.6466	-0.1226	0.2744	0.3429	-0.2231	0	0.3878	-0.6419		1.084	0.2877	0	0.1671	0.4353
T	0.0572	0.1252	-0.1252	-0.2877	-0.3747	-0.8109	0	0.6665		0.2007	-0.1054	0.47	0.3795	-0.3483
V	0.0741	0.08	0.1054	0.1542	-0.1542	-0.2877	0.1625	1.2705		-1.8971	0.2719	-0.0645	-0.2076	0.0645
W	-0.2877	0.4055	-0.9163	0.4055	0.1823	-0.4055	-1.0986	-0.6931		-0.4055	-0.6931	-2.1972	-0.6931	0
Y	-0.8109	0	-1.3218	-0.6931	0	-0.8109	-0.3365	-0.6061		-0.1335	0	-1.0986	-0.1335	0.1178

Color Key

>2	2>	1.5>	1>	0.5 >		> -0.5	> -1	> -1.5	> -2	-2>

Positive scores **0** **Negative scores**
