# Supplementary figures and images for: Developing a powerful In Silico tool for the discovery of novel caspase-3 substrates: a preliminary screening of the human proteome
Source: BMC Bioinformatics. 2012 Jan 23;13:14. doi: 10.1186/1471-2105-13-14 (PMC3324375; doi:10.1186/1471-2105-13-14)

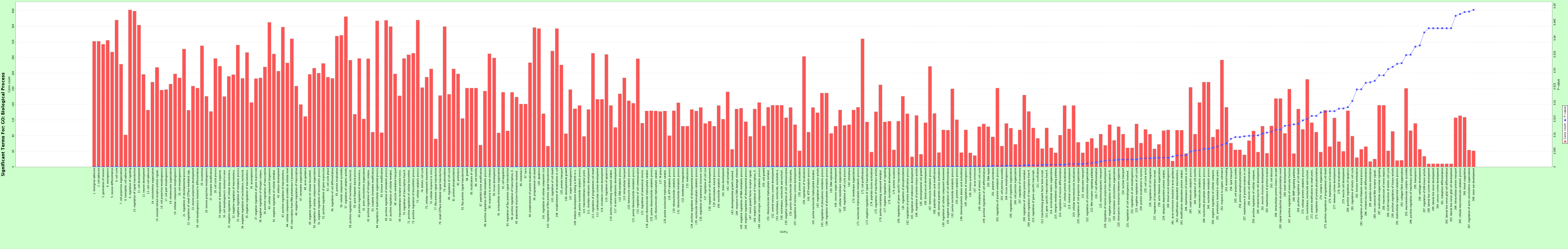

Supplement: Additional file 7 — Chart representing biological processes among predicted substrates. This figure shows the graphical representation of the enriched GO terms: Biological Process among the 3013 predicted caspase-3 substrates. The red bars indicate the gene count (Y-axis) per each GO term: Biological Process (X-axis). The blue dotted line shows the p-value for each GO term that was derived by random sampling from the whole genome analysis. [file 1471-2105-13-14-S7.PNG]
